# Substitution of amino acid residue V1213 in the helicase domain of the genotype 3 hepatitis E virus reduces virus replication

**DOI:** 10.1186/s12985-018-0943-5

**Published:** 2018-02-08

**Authors:** Dianjun Cao, Yan-Yan Ni, Xiang-Jin Meng

**Affiliations:** Department of Biomedical Sciences and Pathobiology, Virginia-Maryland College of Veterinary Medicine, Virginia Polytechnic Institute and State University, 1981 Kraft Drive, Blacksburg, VA 24061-0913 USA

**Keywords:** Hepatitis E virus (HEV), Replication efficiency, Replicon, Pathogenicity

## Abstract

**Background:**

Genotype 3 hepatitis E virus (HEV) infection is generally associated with mild disease. However, recently eight genotype 3 HEV isolates were identified from patients with severe hepatitis. Importantly, three mutations (S605P, I978V and V1213A) in these genotype 3 isolates were found to be typical of genotype 4 HEV, which is sometime associated with more severe hepatitis. Therefore in this study we seek to determine if these unique mutations contribute to enhanced virus replication and thus potentially severe disease.

**Methods:**

In the lack of an efficient cell culture system to study the effect of mutations on HEV replication, we developed a genotype 3 HEV replicon with Renilla luciferase (Rluc) as reporter and subsequently used it to construct numerous mutants, including swMu-1 (V1213A), swMu-2 (Q1246H), swMu-3 (V1213A and Q1246H), swMu-4 (S605P and I978V), and swMu-5 (V1213A, S605P and I978V). RNA transcripts from mutant replicons were transfected into Huh7 S10–3 liver cells to measure the effect of mutations on HEV replication efficiency.

**Results:**

The results showed that the V1213A mutant had the highest reduction in HEV replication efficiency than other mutants. The V1213A and S605P + I978V mutations have a cumulative, if not synergistic, effect on HEV replication. The Q1246H mutant decreased HEV replication compared to the wild-type HEV Rluc replicon but replicated better than the V1213A mutant. The amino acid residue V1213 favors the replication of both genotypes 3 and 4 HEV strains, but not genotype 1 HEV.

**Conclusion:**

The results suggested that the V1213A mutation reduced HEV replication, but is likely not associated with the reported severe hepatitis caused by genotype 3 HEV isolates containing this mutation.

## Background

Hepatitis E virus (HEV) typically causes a self-limiting acute viral hepatitis with large outbreaks reported in individuals from developing countries, and sporadic and cluster cases in individuals from industrialized countries [1, 2]. More recently, chronic hepatitis E has become a significant clinical problem in immunocompromised individuals such as organ transplant recipients [3]. HEV is a single-stranded, positive-sense RNA virus belonging to the family *Hepeviridae* [4], which consists of two genera (*Orthohepevirus,* and *Piscihepevirus*) and five species. Within the species of *Orthohepevirus* A, there are at least 7 genotypes: genotypes 1 and 2 are restricted to humans whereas genotypes 3 and 4 can cross species barriers infecting humans and several other animal species, and genotype 4 HEV is sometimes reportedly associated with severe acute hepatitis in humans [5]. The genotypes 5 and 6 infect wild boar, and genotype 7 infects camels [4]. The pig is a major animal reservoir for zoonotic transmission of HEV to humans [6]. Indeed, sporadic and cluster cases of acute hepatitis E are caused predominantly by the zoonotic genotypes 3 and 4 HEV strains [6, 7].

The majority of the approximately 20 million HEV infections occurred each year worldwide [8] are asymptomatic, as only about 3 millions of these infections actually resulted in clinical cases of viral hepatitis [9]. The mechanisms underlying the induction of liver damage by HEV remains unclear. It has been reported that the genotypes of HEV appear to be associated with disease-inducing potential [10]. Among the 4 major genotypes of HEV that are known to infect humans, the zoonotic genotypes 3 and 4 HEV isolates are distributed worldwide and have been implicated in sporadic cases of acute hepatitis E in humans [1, 6, 9]. Interestingly, fulminant or severe acute hepatitis was reported more frequently in humans infected with genotype 4 HEV in Japan [5, 11] and France [12]. Therefore, the virus genotype of the infected patient may potentially influence the severity of liver disease. However, the genetic element(s) in viral genome that are responsible for viral replication and pathogenesis remain unknown.

The genotype 3 HEV is distributed worldwide, and infects humans, pigs, deer, rabbits and other animal species. Recently, increased virulence associated with HEV genotype 3 (JIO strains) infection was reported from patients with severe hepatitis in Japan, although the course of genotype 3 HEV infection is generally asymptomatic [13]. These JIO strains clustered together with 5 swine isolates from Japan [14] and shared an approximately 98% to 99.8% nucleotide sequence identity with that of swine HEV, suggesting that these apparently “high virulent strains” of genotype 3 HEV may be of zoonotic origin. There is no reported recombination between the JIO strains of genotype 3 HEV and isolates of genotype 4 HEV, but 18 unique amino acid substitutions were identified. Interestingly, three of these mutations (S605P, I978V, and V1213A) located in the helicase or protease domain of HEV were found to be typical of genotype 4 viruses which are sometimes associated with more severe hepatitis. Therefore, it is logical to hypothesize that these mutations may be responsible for the increased virulence reported in these genotype 3 HEV strains in humans. Knowing the effects of these mutations on HEV replication will greatly help us understand the mechanism of HEV pathogenesis. Thus, in this study, we determined the effect of the V1213A, S605P, and I978V amino acid residue mutations on the efficiency of HEV replication using the HEV replicon systems, since currently there is a lack of an efficient cell culture system for HEV propagation.

## Methods

### Cell cultures, HEV infectious clones, and HEV replicons

The Huh7-S10–3 liver cell line that supports a limited level of HEV replication and the genotype 1 human HEV infectious clone (Sar55 strain) are gifts from Dr. Sue Emerson (NIH, Bethesda, MD). The Huh7-S10–3 liver cells were cultured in DMEM medium containing 10% heat-inactivated fetal bovine serum (FBS) at 37 °C in a 5% CO_2_ incubator. The infectious cDNA clones pSHEV3 (genotype 3 swine HEV) and TW9616 (genotype 4 human HEV) were constructed in our laboratory and described previously [15, 16]. The EGFP replicons of genotype 1 HEV Sar55 and genotype 3 HEV pSHEV3 were constructed previously in our laboratory [17]. The pSK-HEV2-hRluc replicon was described previously [18].

### Construction of genotype 3 HEV, and genotype 4 HEV Renilla luciferase (hRluc) replicons

**Fig. 1:**
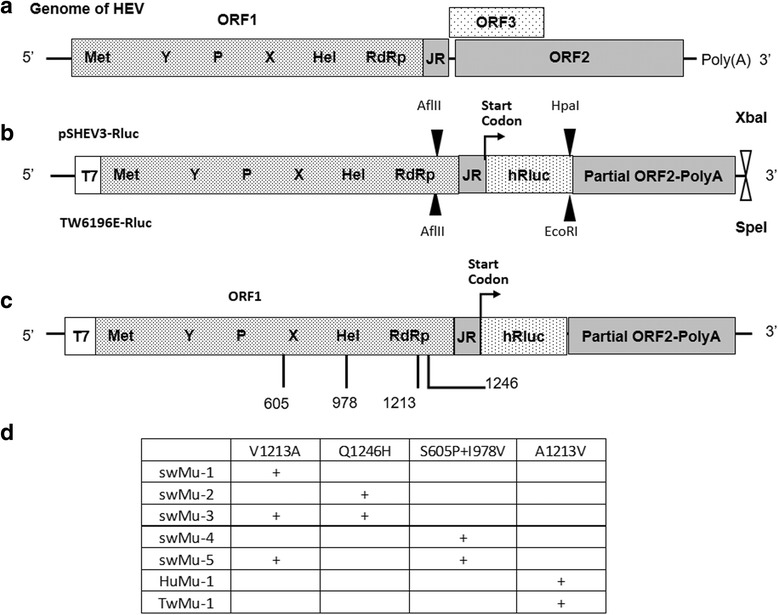
**a** Orgnaization of the HEV genome. **b** A schematic diagram of genotype 3 HEV replicon (pSHEV3-Rluc) and genotype 4 HEV replicon (TW6196E-Rluc). The ORFs and reporter gene hRluc were labeled and showed as boxes. The location of recognition sites of restriction enzymes used for the insertion of hRluc and linearization of replicons were marked on the top of the boxes (pSHEV3-Rluc) and bottom of boxes (TW6196E-Rluc), respectively. **c** The location of mutations on HEV Rluc replicons. The ORF1 was labeled on the top of the box, and the putative ORF1 domains were indicated inside the box. Met, methyltransferase; Y, Y domain; P, a papain-like cysteine protease; X, macro domain; Hel, helicase; RdRp, RNA-dependent RNA polymerase. The amino acid substitutions were labeled as the amino acid residue position (corresponds to the position in genotype 3 HEV strain “US2”) below the ORF1. The Rluc and partial ORF2 were labeled inside boxes. **d** A summary of the HEV mutant Rluc replicons. +: indicates that the mutation created in the replicon

HEV does not replicate efficiently in cell culture, and therefore in order to study the effect of mutations on HEV replication, an HEV replicon system is needed to more robustly assess the effect of mutations on virus replication efficiency. The infectious cDNA clones of the pSHEV3 and TW6196E were used as the backbone for the construction of genotype3 and 4 HEV Rluc replicons, respectively. The Rluc replicons were constructed by synthesizing a PCR fusion product containing the 3’ ORF1 region of pSHEV3 or TW6196E fused to the Rluc gene and substituting it for the *Afl*II to *Hpa*I region in the pSHEV3 infectious cDNA clone or the *Afl*II to *EcoR*I region in the TW6196E infectious cDNA clone (Fig. 1a and b). The 3’ ORF1 region from pSHEV3 or TW6196E was amplified from the infectious cDNA clones by using the primer set SWHEV4623U and SWHEV5208L or TW4675F and TW5168R, respectively (Table 1). The hRluc gene was amplified from pGL4.70[hRluc] vector (Promega) by using the primer set SWhRluc1U and SWhRluc915L or TWJRhRlucU and TWhRlucL, respectively (Table 1). All amplified PCR products were purified with a wizard SV gel and PCR clean-up system (Promega). Subsequently, the pSHEV3- and the TW6196E-related PCR products were mixed, respectively, and the fusion PCR reaction performed. The fusion product was purified by wizard SV gel and PCR clean-up system, digested with *AflI*I-*Hpa*I or *Afl*II-*EcoR*I accordingly, and ligated into the large fragment of the corresponding infectious cDNA clones from which the *Afl*II-*Hpa*I or *Afl*II*-EcoR*I region were deleted (Fig 1b). The inserts of both replicons were confirmed by Sanger DNA sequencing.

**Table 1:** Primers used in the generation of HEV Rluc replicons and their mutants

Primer IDs		Primer Sequence^a^	Modifications^b^
Replicon			
	TW4675F	5’ GCTATGAGTTCCGGGACCTCAA 3’	TW6196-Rluc
	TW5168R	5’ GGTGGCATCGCCATGCAA 3’	
	TWJRhRlucU	5’ TTGCATGGCGATGCCACC ATGGCTTCCAAGGTGTACGAC 3′	
	TWhRlucL	5’ AGAATTCAGCGCTGCTAGCTATTACTGCTCGTTCTTCAGCACG 3’	
	SWhRluc1U	5’ TGCATCGCCCATGGGATCACCATGGCTTCCAAGGTGTACGAC 3’	pSHEV3-Rluc
	SWhRluc915L	5’ CGGTTAACATATGCTAGCCTATTACTGCTCGTTCTTCAGCACG 3’	
	SWHEV4623U	5’ CGCCGAAGGAGTCTCTTAAGGGT 3’	
	SWHEV5208L	5’ GTCGTACACCTTGGAAGCCATGGTGATCCCATGGGCGATGCA 3’	
Mutagenic			
	HEV3MvaU	5’ GTGAGGTCGGCATTTCGGATGCAATTGTCAATAACTTTTTCCTTG 3’	V1213A
	HEV3MvaL	5’ CAAGGAAAAAGTTATTGACAATTGCATCCGAAATGCCGACCTCAC 3’	
	HEV3MqhU	5’ GATCAGAACCTCGGGACTCTACATGCCTTCCCGCCGTCCTGCCAG 3′	Q/H
	HEV3MqhL	5’ CTGGCAGGACGGCGGGAAGGCATGTAGAGTCCCGAGGTTCTGATC 3’	
	HEV3MspU	5’ GTCTATGGGGGCCGGGCCTCATAGCCTCACTTATG 3’	S605P
	HEV3MspL	5’ CATAAGTGAGGCTATGAGGCCCGGCCCCCATAGAC 3’	
	HEV3MivU	5’ GCTTGTGCCGGCTGCACTGTTAGTCCTGGGATTG 3′	I978V
	HEV3MivL	5’ CAATCCCAGGACTAACAGTGCAGCCGGCACAAGC 3’	
	HEV3MvaHU	5’ GTGAGGTCGGCATTTCGGATGCTATTGTCAATAACTTTTTCCTTG 3’	V1213A (2nd)
	TWMavU	5’ TGAGGTGGGTATCTCTGATGTTATTGTTAACAACTTCTTCC 3′	A1213V TW6196
	TWMavL	5’ GGAAGAAGTTGTTAACAATAACATCAGAGATACCCACCTCA 3′	
	HEV2MavU	5’ GCGAGGTGGGCATCTCCGATGTGATCGTTAATAACTTTTTCCTTG 3’	A1213V Sar55
	HEV2MavL	5’ CAAGGAAAAAGTTATTAACGATCACATCGGAGATGCCCACCTCGC 3’	

^a^Sequences of primers

^b^Rluc inserted into HEV genomes or amino acid mutation introduced by the primer

### Construction of mutants of genotype 3 HEV replicon

The mutants containing the V1213A mutation in ORF1 of HEV genotype 3 GFP replicon (pSHEV3-EGFP) and the A1213V mutation in ORF1 of HEV genotype 1 GFP replicon (pSK-HEV2-EGFP) were constructed with QuikChange® II XL Site-Directed Mutagenesis Kit (Stratagene). Using the pSHEV3-Rluc replicon as the backbone, we also constructed 5 mutants with mutations in the ORF1, designated as (1) swMu-1 mutant containing the V1213A mutation; (2) swMu-2 mutant containing the Q1246H mutation; (3) swMu-3 mutant containing the Q1246 + V1213A mutations; (4) swMu-4 mutant containing S605P + I978V mutations, and (5) swMu-5 mutant containing S605P + I978V + V1213A mutations (Fig. 1c and d). The mutants containing single site mutation were generated with QuikChange® II XL Site-Directed Mutagenesis Kit (Stratagene) and the mutants containing double or triple mutations were produced with QuikChange® multi Site-Directed Mutagenesis Kit (Stratagene) according to the manufacturer’s instructions. All primers used to introduce the mutations were listed in Table 1.

### Construction of mutants of genotype 1 and genotype 4 HEV Rluc replicons

To assess the impact of amino acid substitution at position 1213 in the ORF1 on virus replication of genotype 1 and genotype 4 HEV, we constructed the A1213V mutant replicon in the HEV hRluc replicon of HEV genotype 1 (pSK-HEV2-Rluc) and genotype 4 (TW6196-Rluc), respectively, with QuikChange® II XL Site-Directed Mutagenesis Kit (Stratagene). The TW6196-Rluc HEV replicon was constructed in this study, and the primers used for the construction were listed in Table 1.

### In vitro RNA transcription and transfection

The plasmids containing the backbone of pSK-HEV2 (genotype 1 HEV) were linearized with *Bgl*II. The plasmids containing the backbone of pSHEV3 (genotype 3 HEV) were digested with *Xba*I, and the plasmids containing genotype 4 HEV backbone were linearized with *Spe*I. The purified linearized plasmid DNA was used as templates for in vitro transcription of capped RNA with the mMessage mMachine T7 ultra kit (Ambion) as described previously [18].

The capped RNA transcripts from each of the HEV replicons as well as replicons containing mutation(s) were co-transfected together with firefly luciferase RNA into the Huh7-S10–3 liver cells with DMRIE-C reagent (Invitrogen) following the manufacturer’s instruction. Huh7-S-10-3 cells that had been transfected with RNA transcripts from HEV replicons or mutants were harvested at 5 days post-transfection. The GFP expression signals were analyzed by flow cytometry as described previously [19]. The luciferase activities were measured with a dual luciferase reporter assay system (Promega), and firefly luciferase signals from the co-transfected firefly luciferase RNA were used to normalize the Renilla luciferase signals [17, 18]. Relative Luminescence Unit (RLU) is measured at 5 days post-transfection and normalized with the signal from the co-transfected firefly luciferase RNA. Data were collected from an average of eight separate replicate experiments. For the comparison of RLU produced by multiple mutants of HEV Rluc replicons, data were presented as relative luciferase activities to wild-type replicon (the ration of the sample’s RLU to that produced by the wild-type replicon), and the RLU for the wild-type Rluc replicons were set as 100%.

### Statistical analysis

Multiple comparisons among the experimental groups were analyzed with one-way analysis of variance (ANOVA) followed with Kruskal-wallis test, and the comparisons between two groups were calculated with two-tailed *t*-test. All data analyses were performed with GraphPad Prism 6 (GraphPad Software, Inc.). The significant difference was defined as *P* < 0.05, and the highly significant difference as *p* < 0.01.

## Results

### The A1213V mutation in genotype 1 HEV and the V1213A mutation in genotype 3 HEV decreased virus replication efficiency

**Fig. 2:**
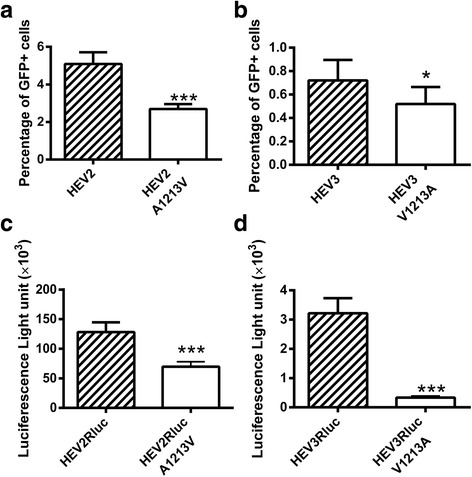
Effect of mutation at amino acid residue 1213 on genotype 1 and genotype 3 HEV replicons. **a** A1213V mutation on genotype 1 HEV EGFP replicon. HEV2, pSKHEV2-EGFP replicon; HEV2 A1213V, pSKHEV2-EGFP replicon with mutation A1213V. **b** V1213A mutation on genotype 3 HEV EGFP replicon. HEV3, pSHEV3-EGFP replicon; HEV3 V1213A, pSHEV3-EGFP replicon with mutation V1213A. **c** A1213V mutation on genotype 1 HEV luciferase replicon. HEV2Rluc, pSKHEV2-Rluc replicon; HEV2Rluc A1213V, pSKHEV2-Rluc replicon with mutation A1213V. **d** V1213A mutation on genotype 3 HEV luciferase replicon. HEV3Rluc, pSHEV3-Rluc replicon; HEV3Rluc V1213A, pSHEV3-Rluc replicon with mutation V1213A. The error bars indicate standard deviations (SD). The differences of RLU produced by HEV Rluc mutants and the wild-type Rluc replicon were compared by *t-*test. Asterisks (*) indicate statistical differences compared to the wild-type HEV replicons. *, *P* < 0.05; ***, *P* < 0.001

To determine if the mutation at amino acid residue 1213 in genotypes 1 and 3 HEV has any effect on virus replication, we compared the replication levels of HEV EGFP replicons containing the A1213V mutation in genotype 1 and V1213A mutation in genotype 3 HEV replicons. A significantly lower number of GFP-positive cells was observed in Huh7 S10–3 liver cells transfected with the genotype 1 pSK-HEV2-EGFP replicon containing the A1213V mutation (Fig 2a). The V1213A mutation in the genotype 3 HEV EGFP replicon showed a slightly but significant lower number of EGFP-positive Huh7 S10–3 cells as well (Fig 2b). The results suggest that the mutation at amino acid residue 1213 reduced the replication level of both genotype 1 and genotype 3 HEV replicons.

However, we found that the EGFP HEV replicon systems are not very sensitive for measuring HEV replication level. For example, the genotype 3 HEV EGFP replicon not only showed lower number of EGFP-positive cells in the transfected cells but also exhibited a lower density of GFP signal in the positive cells. Since both the number of positive cells and the density of positive signals are related to the replication level of HEV replicons, a more sensitive replicon system such as Renilla luciferase replicon is needed to compare the replication level of HEV and their mutants in the absence of an efficient cell culture system for HEV propagation.

### HEV luciferase replicon system reflects virus replication more accurately than the HEV EGFP replicon

By using a Renilla luciferase as the reporter, we developed HEV genotypes 1 and 3 Rluc replicon systems. Subsequently, a V1213A mutant Rluc replicon (swMu-1) was constructed in the HEV genotype 3 Rluc replicon, and a A1213V mutant was constructed in the HEV genotype 1 Rluc replicon. The luciferase activities in Huh7 liver cells transfected with the RNAs from the V1213A and A1213V mutant replicons were measured and analyzed. The results showed that the A1213V mutation in genotype 1 HEV Rluc-replicon reduced HEV replication level similar to what we found with the EGFP replicon system (Fig 2c). The V1213A mutation in HEV genotype 3 Rluc-replicon reduced HEV replication efficiency more significantly than what we observed with the EGFP replicon system (Fig 2d). The data suggested that the HEV luciferase replicon system is more sensitive, and reflects HEV replication more accurately than HEV EGFP replicon system.

### The S605P and I978V double mutations in genotype 3 HEV significantly decreased virus replication efficiency

**Fig. 3:**
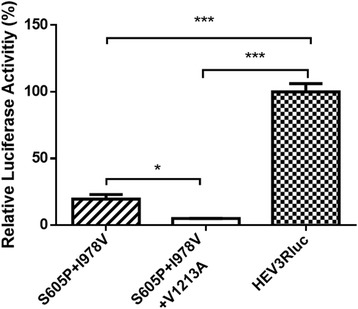
Relative luciferase activities in Huh7 S10–3 liver cells transfected with RNA of HEV genotype 3 Rluc mutants containing S605P + I978V, and V1213A mutations. S605P + I978V: pHEV3-RLuc contains mutations of S605P and I978V; S605P + I978V + V1213A: pHEV3-RLuc contains mutations of S605P, I978V and V1213A. The error bars indicate standard deviations (SD). The differences of relative luciferase activity produced by mutants of HEV Rluc replicons and the wild-type HEV Rluc replicon were compared by one-way analysis of variance (ANOVA) using the Kruskal-Wallis test (*P* < 0.05). Asterisks (*) indicate statistical differences compared to the parental wild-type HEV replicons, *, *P* < 0.05; ***, *P* < 0.001

When mutations S605P and I978V were introduced into the pSHEV3-Rluc genotype 3 HEV replicon, we found that the dual mutations significantly reduced the replication efficiency of pSHEV3-Rluc HEV replicon, which can be further reduced by introducing the V1213A mutation (Fig 3). The results indicated that the V1213A mutation affected the replication efficiency of genotype 3 HEV more significantly than S605P + I978V mutations. To confirm that the effect of V1213A mutation on HEV replication was caused by amino acid change, not the nucleic acid, we constructed another V1213A mutation with a different condon, GCA, in place of the previous codon GCT. There was no difference on the efficiency of HEV replication when S650P + I978V replicon was combined with different codons of the V1213A mutation, suggesting that the effect of the mutation on HEV replication was caused by the amino acid change.

### The Q1246H mutation in genotype 3 HEV reduced virus replication efficiency at a lower level than the V1213A mutation

**Fig. 4:**
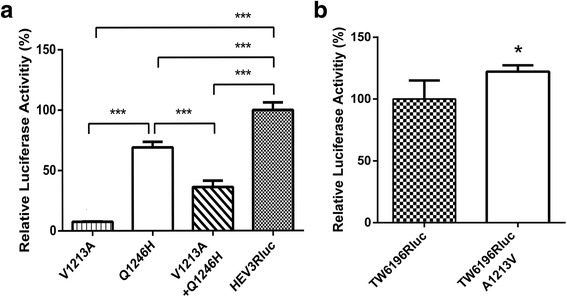
Relative luciferase activities in Huh7 S10–3 liver cells transfected with (**a**) RNA of HEV Rluc mutants containing Q1246H and V1213A in HEV genotype 3 Rluc replicons, and (**b**) RNA of A1213V mutation in genotype 4 Rluc replion. V1213A: mutation of V1213A; Q1246H: mutation of Q1246H; V1213A + Q1246H: mutation of Q1246H and V1213A; A1213V: mutation of A1213V. are measured at 5 days post-transfection and normalized with co-transfected firefly luciferase RNA. The error bars indicate standard deviations (SD). *, *P* < 0.05; **, 0.05 < *P* < 0.01; ***, *P* < 0.001

In addition to the mutations of V1213A, S605P and I978V, there is also a Q1246H mutation identified in the high virulent genotype 3 HEV isolates, but the H residue is different from the corresponding residue typically found in the genotype 4 isolates [13]. To determine the effect of Q1246H mutation found in the high virulent genotype 3 HEV isolates on the virus replication, a panel of HEV mutants were also constructed in HEV Rluc-replicon to determine if these unique amino acid mutations affect HEV replication efficiency. In the genotype 3 HEV Rluc replicon system, we found that the V1213A mutation reduced HEV replication efficiency more significantly than the Q1246H mutation (Fig 4a). The V1213A + Q1246H double mutations had a more significant reduction of HEV replication level than the Q1246H mutation alone (Fig 4a). The results suggested that the V1213A mutation is more important to the replication of genotype 3 HEV than the Q1246H mutation, and that there was no apparent synergistic effect between V1213A mutation and Q1246H mutation.

### The A1213V mutation in genotype 4 HEV significantly increased virus replication efficiency

Furthermore, to determine if the function of amino acid residue at position 1213 is HEV genotype-specific, we constructed a A1213V mutant in the genotype 4 HEV Rluc-replicon. Contrary to what we observed with genotype 1 HEV, we found that the A1213V mutation significantly increased the replication level of genotype 4 HEV (Fig 4b). The results suggested that the amino acid residue V at position 1213 actually favors the replication of both zoonotic genotypes 3 and 4, but not genotype 1.

## Discussion

Genotype 3 HEV infection typically is associated with asymptomatic or mild disease, although in immunocompromised individuals genotype 3 HEV causes chronic infection [3]. However, there was a report in Japan [13] of severe hepatitis associated with 8 genotype 3 HEV isolates. Interestingly, three unique mutations (S605P, I978V and V1213A) identified in the ORF1 of these genotype 3 isolates were found to be characteristic of genotype 4 HEV strains, which was considered to be more likely associated with severe hepatitis [13]. Due to the lack of a small animal model for HEV pathogenicity study and a robust cell culture system to study HEV replication, the virus replication fitness or efficiency is often used as a convenient and reliable method to estimate if the mutation(s) in viral genome has any impact on the virus virulence. Therefore, in this study, we aimed to determine if these observed mutations in these genotype 3 HEV isolates affect the replication of HEV in vitro.

By using the HEV GFP replicons system, we first demonstrated that the mutation at amino acid residue 1213 reduced the replication efficiency of both HEV genotype 1 (A1213V) and HEV genotype 3 (V1213A). Our results indicated that the amino acid residue 1213 is critical for HEV replication, which is consistent with a previous report [13]. However, the replication level of genotype 3 HEV EGFP replicons was low and not very sensitive, as evidenced by the low number of GFP-positive cells and low density of GFP signal, which makes it difficult to more accurately evaluate the replication efficiency in GFP-positive cells. To circumvent the obstacle of the low efficiency of HEV replication, particularly with the genotype 3 HEV EGFP replicon, we subsequently constructed renilla luciferase HEV replicons, which were proved to be more sensitive and accurately reflect the HEV replication level than the HEV EGFP replicons.

The pSHEV3-Rluc genotype 3 HEV replicon system yielded similar, but more reliable, results than that of the HEV EGFP replicons system, demonstrating that the V1213A mutation significantly reduced the replication efficiency of genotype 3 HEV, whereas the A1213V mutation slightly decreased the replication efficiency of genotype 1 HEV. The amino acid residue 1213 locates in the helicase domain of the HEV ORF1, which has been considered as a critical factor for HEV replication. Since the A1213V mutation in genotype 1 HEV also reduced the viral replication efficiency, suggesting that the V1213 residue is not the optimal residue for genotype 1 HEV replication.

When we examined the effect of other mutations (S605P and I978V) that were characteristic of genotype 4 HEV on virus replication, we found that S605P and I978V mutations significantly reduced the efficiency of genotype 3 HEV replication (*p* < 0.001). Introduction of the V1213A mutation into the S605P-I978V double mutant further reduced the viral replication efficiency (*p* < 0.05), suggesting that the two sets of mutations have a cumulative, if not synergistic, effect on genotype 3 HEV replication. To further verify that the effect of mutation V1213A is caused by the amino acid, we used a different synonymous codon of the Alanine to create the V1213A mutation in the S605P + I978V mutant, and similar results in HEV replication efficiency were obtained. Taken together, the data suggested, contrary to what we initially thought, that the three mutations identified in the genotype 3 HEV isolates associated with severe hepatitis in Japan actually decreased the efficiency of replication of genotype 3 HEV. The S605P and I978V mutations are located in a relatively high variable region of the ORF1 protein, whereas the V1213A mutation is located in the helicase, an enzyme involving in viral replication. Interestingly, here we demonstrated that the Q1246H mutation identified in the high virulent genotype 3 HEV isolates, but the H residue is different from the corresponding residue typically found in the genotype 4 isolates, only decreased the replication efficiency of genotype 3 HEV at a lower level as compared to that of the V1213A mutation. Our results revealed that the V1213A mutation affects HEV replication efficiency more than that of Q1246H mutation. The V1213A mutation has more drastic effect on HEV replication efficiency, and this may likely due to the effect of the mutation on the function of viral helicase.

Interestingly, when we assessed the effect of A1213V mutation on the replication efficiency of genotype 4 HEV using the genotype 4 HEV luciferase replicon system, we found that the A1213V mutation slightly increased the replication level of genotype 4 HEV (*p* < 0.05). The data suggested that the amino acid residue V1213 favors the replication of both zoonotic HEV genotypes 3 and 4, but not genotype 1 HEV, as compared to amino acid residue A1213. The V1213A mutation found in the patients infected with the genotype 3 virus isolates may reduce HEV replication level, and thus was likely not a key factor responsible for the observed severe hepatitis associated with these genotype 3 isolates containing this mutation. HEV infection causes acute viral hepatitis but can also result in chronic hepatitis E in immunocompromised patients [20]. Genotype 4 HEV was reportedly associated with severe diseases more frequently than genotype 3 HEV [11, 12]. An insertion of a 58 amino acid residues of human ribosomal protein S17 in the hypervariable region (HVR) of HEV ORF1 apparently enhances HEV replication efficiency in vitro [21]. Together with our data from this study, it appears that both host-specific factors and virus genetic element(s), including virus genotype and mutations, may contribute to HEV pathogenicity.

## Conclusion

The results from this study suggested that the V1213A mutation reduced HEV replication, and is not likely associated with the reported severe hepatitis caused by genotype 3 HEV isolates containing this mutation.
